# Supplementary material for: PCGF1-PRC1 links chromatin repression with DNA replication during hematopoietic cell lineage commitment
Source: Nat Commun. 2022 Nov 28;13:7159. doi: 10.1038/s41467-022-34856-8 (PMC9705430; doi:10.1038/s41467-022-34856-8)
Supplement: Supplementary file 1 — Supplementary Information [file 41467_2022_34856_MOESM1_ESM.pdf]

Supplementary Figure. 1

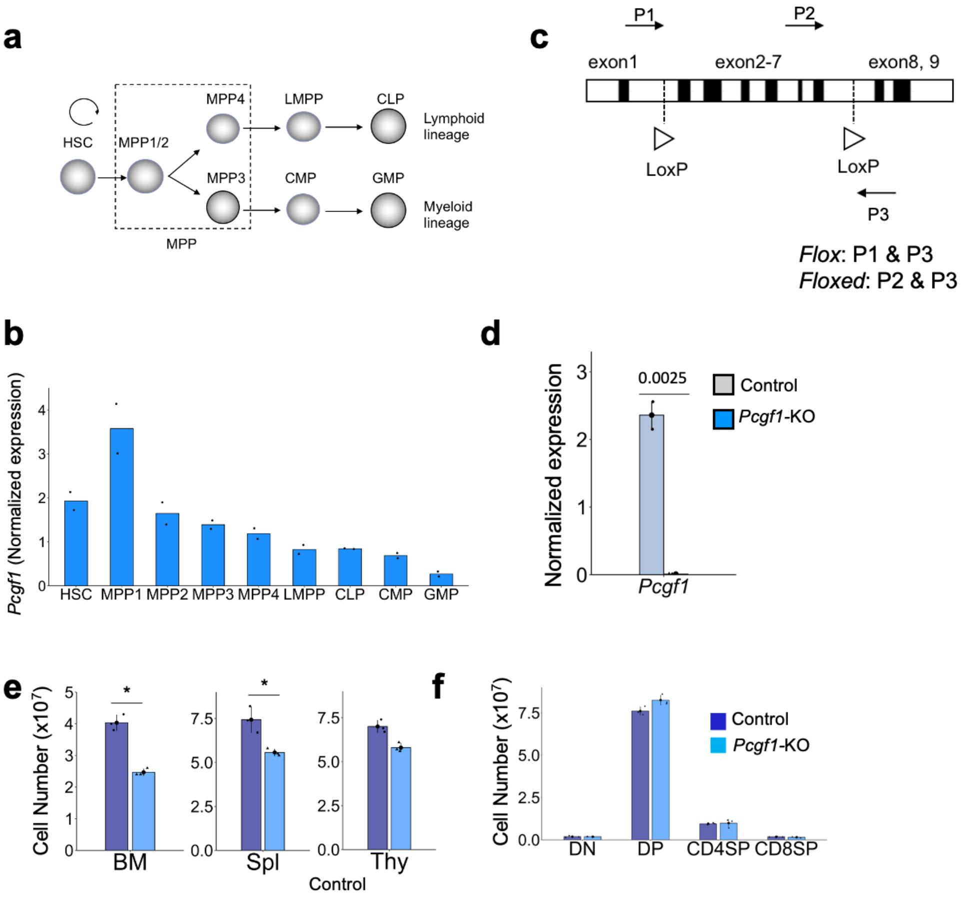

Supplementary Figure. 1 (continued)

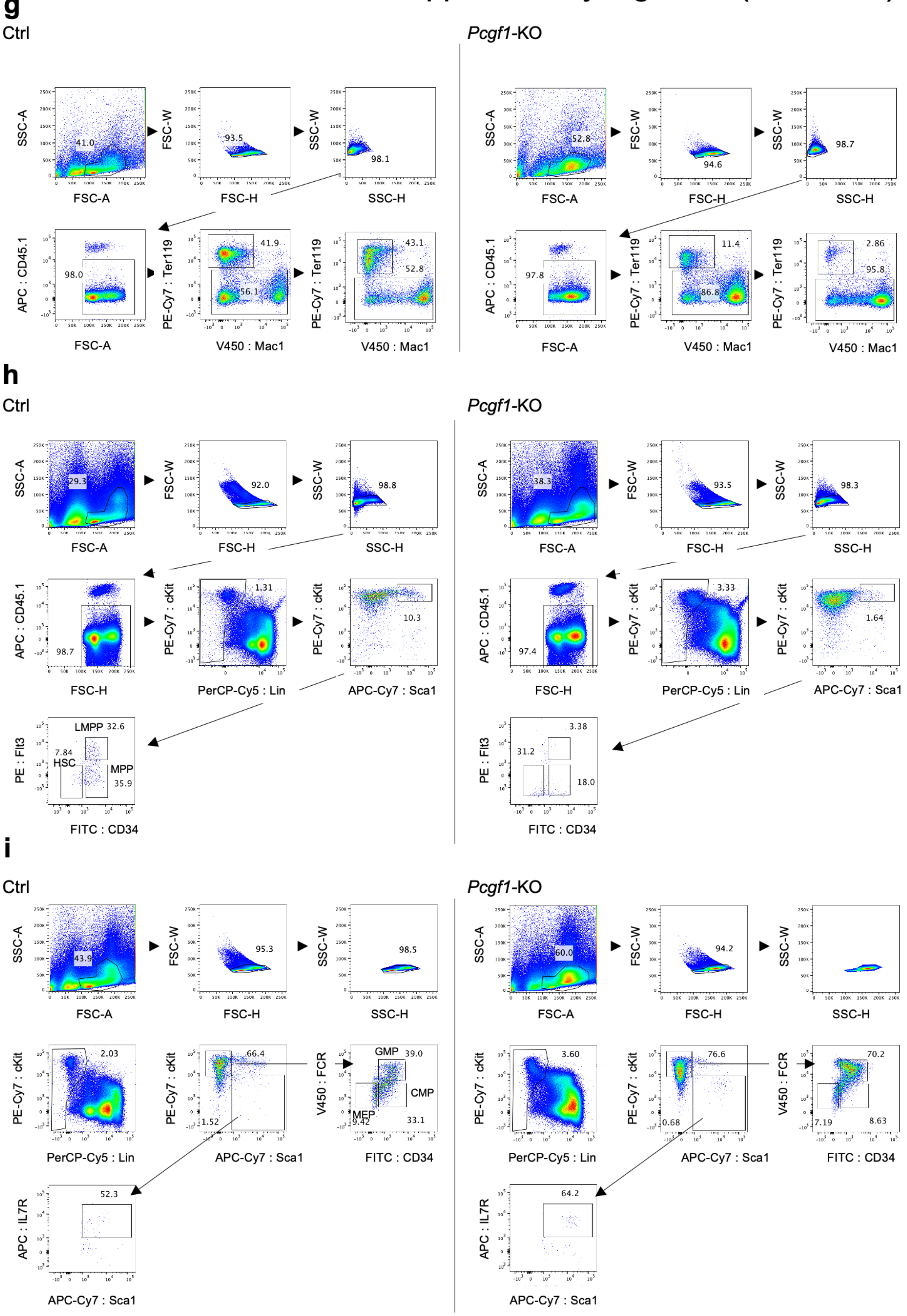

Supplementary Figure. 1 (continued)

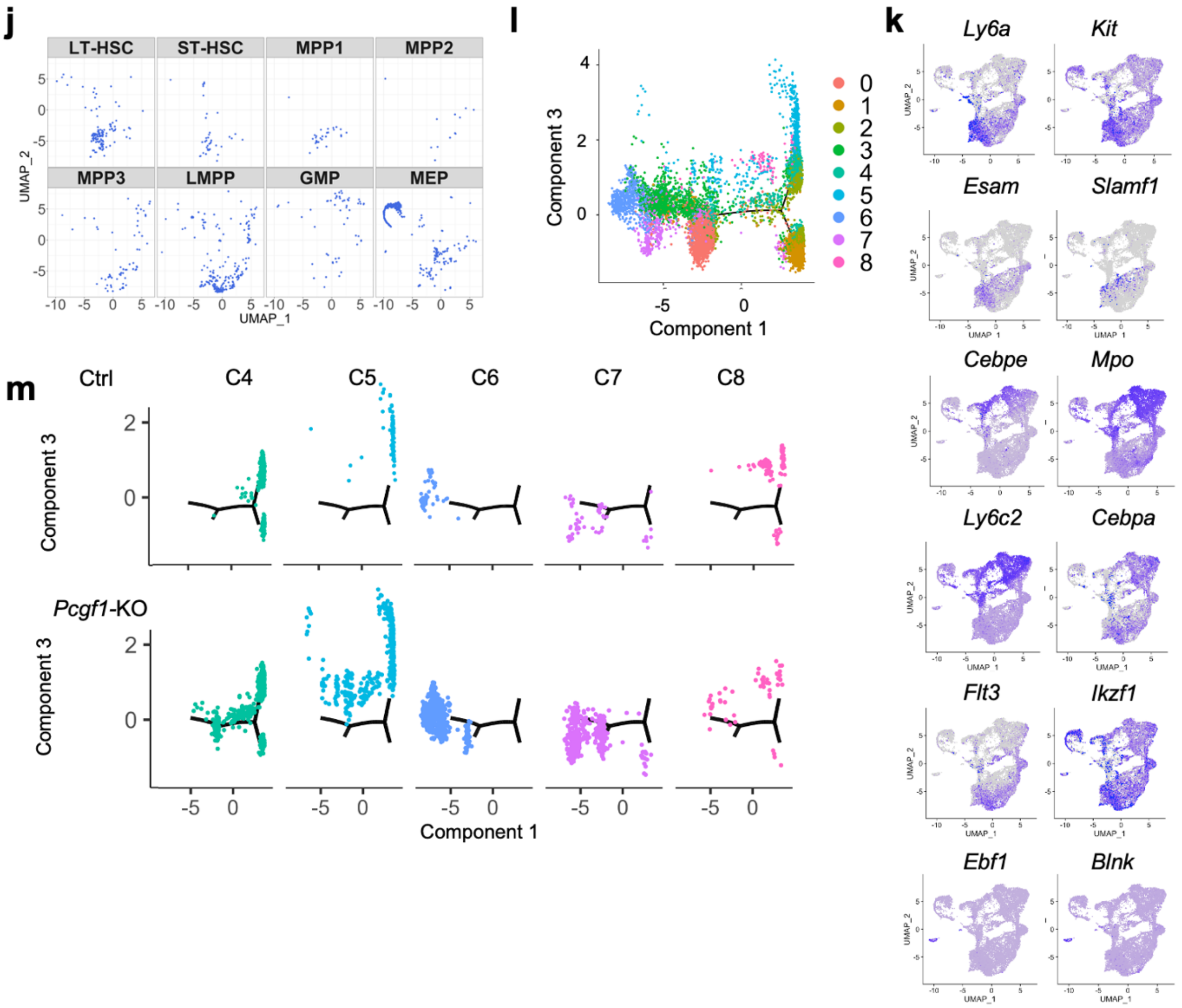

Supplementary Fig. 1 (Related to Fig. 1). Expression of *Pcgf1* during early hematopoiesis and generation of a *Pcgf1* conditional knock out (cKO) allele and *Pcgf1* cKO IdHPCs.

(**a**) Schematic representation of lineage commitment and differentiation of HSCs. (**b**) Normalized expression of *Pcgf1* revealed by qRT-PCR in the indicated cell types is shown. Data represent the mean of two independent qRT-PCR experiments. (**c**) Schematic representation of the gene targeting strategy to generate a conditional KO allele of *Pcgf1*. Two loxP sites were inserted to enable induced deletion of a genomic region encompassing exons 2 to 7. Genomic PCR primers used for detection of the WT and KO alleles are indicated. The neomycin-resistant gene cassette (Neo) was deleted after germline transmission of the mutant allele by mating with FRT-expressing transgenic mice. (**d**) *Pcgf1* expression in LMPP cells derived from *Pcgf1*$^{fl/fl}$ or ERT2-Cre;*Pcgf1*$^{fl/fl}$ mice treated with 4-OHT analyzed by RT-qPCR. FACS sorted LMPP cells were cultured for 4 days with 4-OHT using TSt-4 stromal cells and media supplemented with SCF, IL-7 and Flt3-L. Data represent the mean±SD of three biologically independent qRT-PCR experiments. The numbers on the graph are *p*-values between the control and *Pcgf1-KO* calculated with the Welch's two-sided *t* test. (**e**) Defects in repopulation of *Pcgf1*-KO hematopoietic cells in BM and spleen (Spl) but not in thymus (Thy). Total numbers of graft-derived cells in the respective tissues were compared between *Pcgf1*-KO and the control (Ctrl). Data represent the mean±SD of three biologically independent experiments. *$p<0.05$; (Welch's two-sided *t* test). *P*-values are 0.003 (BM) and 0.04 (Spl). (**f**) Numbers of CD4$^-$CD8$^-$ (DN), CD4$^+$CD8$^+$ (DP), CD4$^+$CD8$^-$ (CD4SP) and CD4$^-$CD8$^+$ (CD8SP) thymocytes in control and *Pcgf1*-KO. Data represent the mean±SD of three biologically independent experiments. **(g)(h)(i)** Representative flow cytometric profiles of graft-derived BM cells from *Pcgf1*-KO or the control (Ctrl) to identify the respective cell types. Surface antigens and cell fractions tested are CD19 and Mac1 in CD45.1$^-$Ter119$^-$ cells to identify lymphoid and myeloid

cells (**g**), Flt3 and CD34 in CD45.1$^-$Lin$^-$c-Kit$^+$Sca-1$^+$ cells to identify HSC, MPP and LMPP (**h**), and FCR and CD34 in CD45.1$^-$Lin$^-$c-Kit$^+$Sca-1$^-$ cells to identify CMP, GMP and MEP (**i**). The percentage of the respective cell types indicated by boxes is shown. The data shown in the graphs are representative of three independent biological experiments. (**j**) Distribution of the respective cell types in the Umap plot as shown in this study according to the definitions in Sonia Netrowa's paper[41] (GSE81692). (**k**) The expression of marker genes for HSCs (*Esam, Slamf1*), myeloid progenitors (*Cebpa, Cebpe, MPO, Ly6c2*), lymphoid progenitors (*Flt3, Ikzf1*), and B lineage-primed cells (*Ebf1, Blnk*) in the Umap plot as shown in this study. (**l**)(**m**) An inferred pseudo-time trajectory in all LSKs (**l**) and in each cluster defined in Fig. 1e (**m**) in the control (Ctrl) and *Pcgf1*-KO.

Supplementary Figure. 2

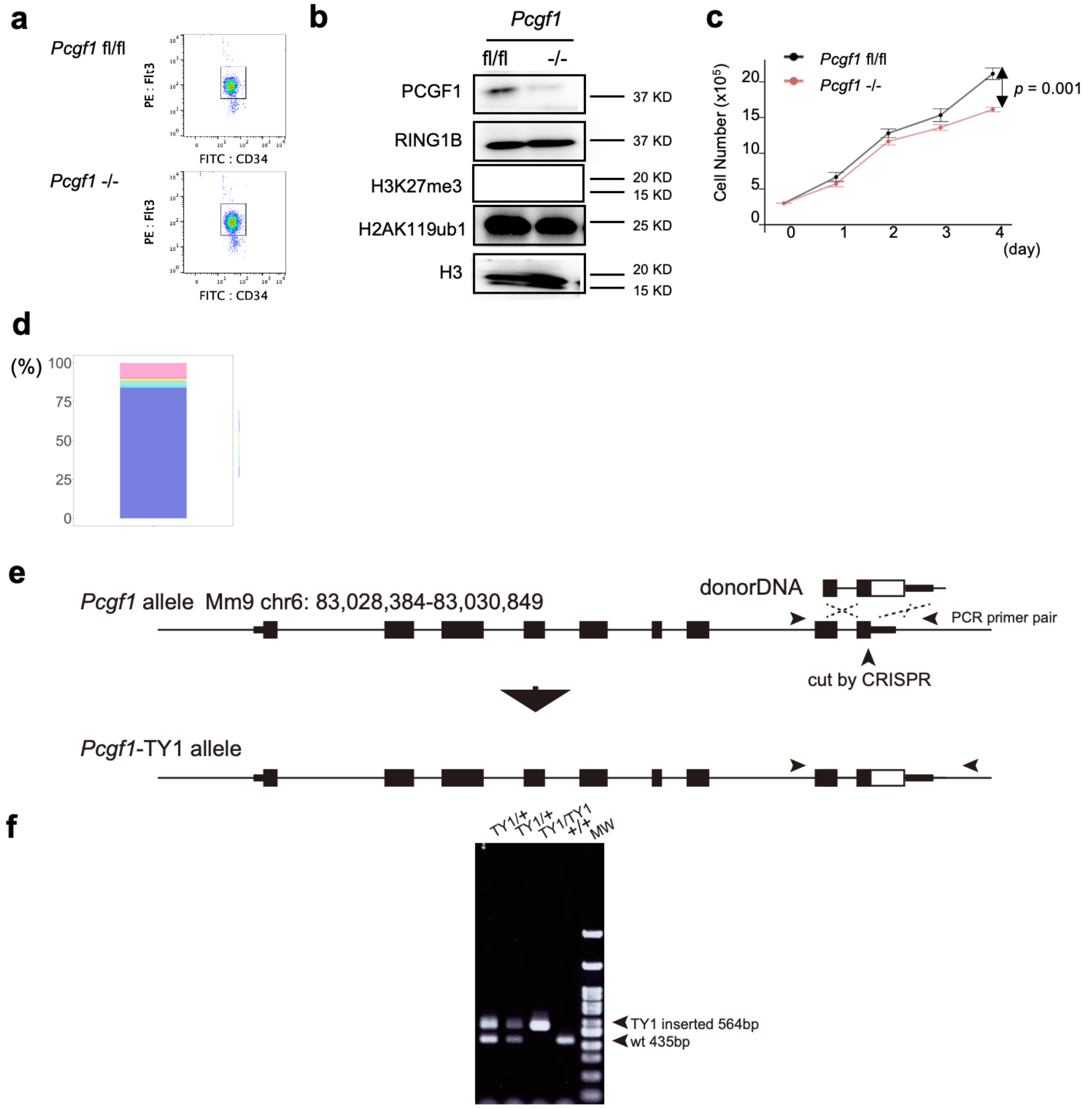

Supplementary Figure. 2 (continued)

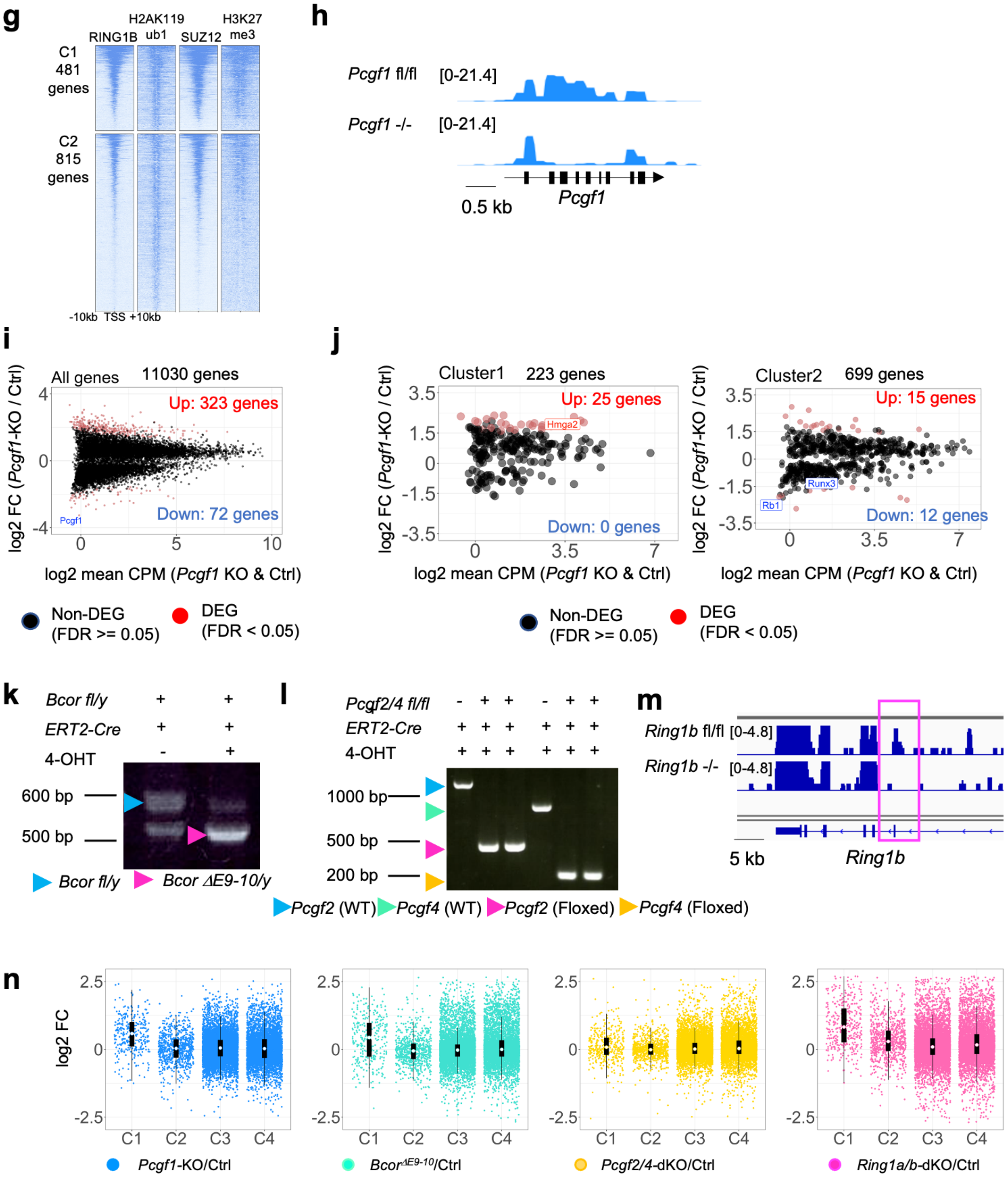

Supplementary Fig. 2 (Related to Fig. 2). PCGF1 downregulates H3K27me3 marked genes in collaboration with RING1A/B.

(**a**) The surface expression of Flt3 and CD34 in control and 4-OHT-treated ERT2-Cre;*Pcgf1*$^{fl/fl}$ IdHPCs. (**b**) Immunoblotting with the indicated antibodies confirmed a significant reduction of *Pcgf1* expression induced by 4-OHT treatment. Data in (**a**) and (**b**) are representative of two independent biological replicates. (**c**) Proliferative capacity of *Pcgf1*-KO IdHP cells. Graphic data shown in (**c**) represent mean ± SD for biological triplicates. The numbers on the graph is the *p*-value between the control and *Pcgf1-KO* calculated with the Welch's two-sided *t* test. (**d**) Predominant distribution of PCGF1 peaks at promoter regions. **(e)** Schematic representation of the strategy to generate a *Pcgf1*-TY1 allele using the Alt-R CRISPR-Cas9 system (IDT) (https://sg.idtdna.com/pages/products/crispr-genome-editing/alt-r-crispr-cas9-system). See materials and methods for more details. **(f)** Genomic PCR confirming the insertion of *Ty1* into the *Pcgf1* locus. The data shown in the graph are representative of two independent biological replicates. (**g**) A heatmap of ChIP-seq data for indicated antibodies in ESCs. (**h**) Genome browser tracks showing the significant reduction of *Pcgf1* expression in *Pcgf1*-KO IdHPCs. (**i**) An MA plot for differentially expressed genes (DEG) between control (*Pcgf1*$^{fl/fl}$) and *Pcgf1*-KO IdHPCs. Log2 fold change is plotted against the mean normalized gene expression of control and *Pcgf1*-KO IdHPCs. FDR was calculated by edgeR. Low abundance genes (CPM < 1 across all samples) were filtered out. DEGs are indicated by red dots. Data in the graph are the averages of two biologically independent experiments. (**j**) MA plots for cluster 1 and 2 genes shown in the same manner as in (**i**). **(k)** Genomic PCR showing the induced deletion of exons 9 and 10 of the *Bcor* gene by 4-OHT treatment in ERT2-Cre;*Bcor*$^{fl/y}$ IdHPCs. **(l)** Genomic PCR showing the induced deletion of *Pcgf2* and *Pcgf4* in ERT2-Cre:*Pcgf2*$^{fl/fl}$:*Pcgf4*$^{fl/fl}$ IdHPCs by 4-OHT treatment. Data in (**k**) and (**l**) are

representative of two independent biological replicates. (**m**) Genome browser tracks showing decreased expression of *Ring1b* in *Ring1a/b* double KO (dKO) IdHPCs. **(n)** A boxplot showing the changes in gene expression in respective mutant IdHPCs. Up-regulation of C1 genes was observed in *Pcgf1*-KO, *Bcor*$^{\Delta E9\text{-}10}$, and *Ring1a/b*-dKO, but not in *Pcgf2/4*-dKO IdHPCs. The center circle indicates a median value and the boxes indicate the 25th to 75th percentile. Each dot represents individual genes. Data in graphs represent the average of two biologically independent analyses.

Supplementary Figure. 3

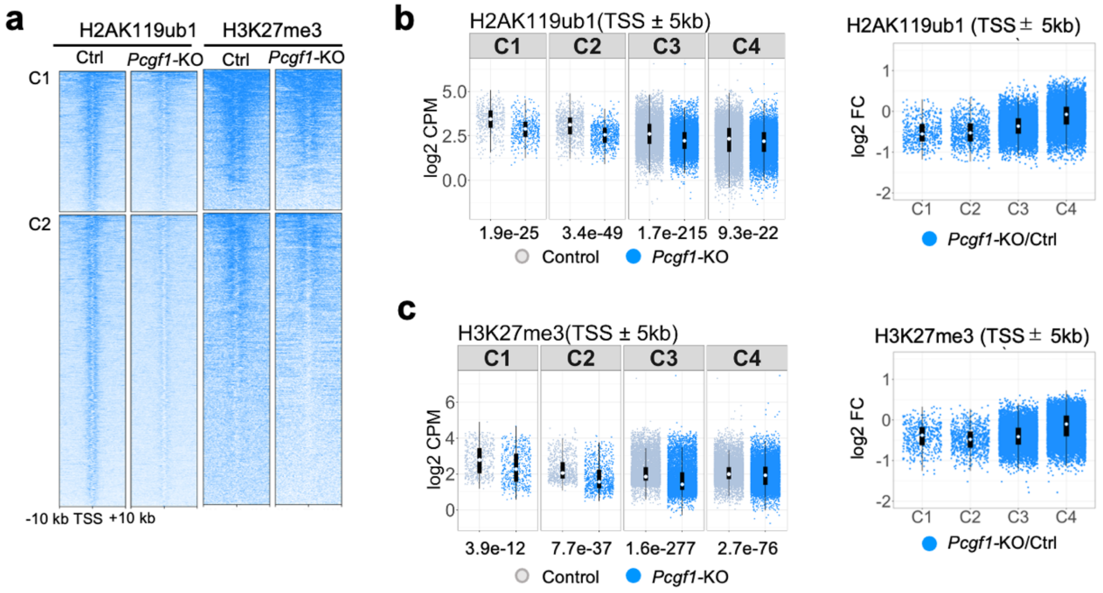

Supplementary Fig. 3 (Related to Fig. 3). *Pcgf1* deletion in ESCs results in destabilization of local H2AK119ub1 in contrast to IdHPCs.

(a)(b)(c) Contribution of PCGF1 to stabilize local enrichment of H2AK119ub1 and H3K27me3 around TSSs in ESCs. **(a)** Heatmap views of ChIP-seq signals for H2AK119ub1 and H3K27me3 across TSS (±10 kb) regions of C1 and C2 genes in control and *Pcgf1*-KO ESCs. Representative data of the two biologically independent analyses are shown. **(h)** Box plots for ChIP-seq signals across TSS (±5kb) for H2AK119ub1 in each cluster in control and *Pcgf1*-KO ESCs (left panel) and their fold changes signals in *Pcgf1*-KO (right panel). Data in graphs represent the means of two biologically independent analyses. The center circle indicates a median value and the boxes indicate the 25th to 75th percentile. Each dot represents individual genes. The numbers beneath the graph are *p*-values between the control and *Pcgf1-KO* calculated with the Wilcoxon signed-rank test in the left panel. CPM: Counts Per Million. **(c)** Box plots for ChIP-seq signals across TSS (±5kb) for H3K27me3 in each cluster in control and *Pcgf1*-KO ESCs (left panel) and their fold changes in *Pcgf1*-KO (right panel). Data in graphs represent means for two biologically independent experiments. The center circle indicates a median value and the boxes indicate the 25th to 75th percentile. Each dot represents individual genes. The numbers beneath the graph are *p*-values between the control and *Pcgf1-KO* calculated with the Wilcoxon signed-rank test in the left panel. CPM: Counts Per Million.

Supplementary Figure. 4

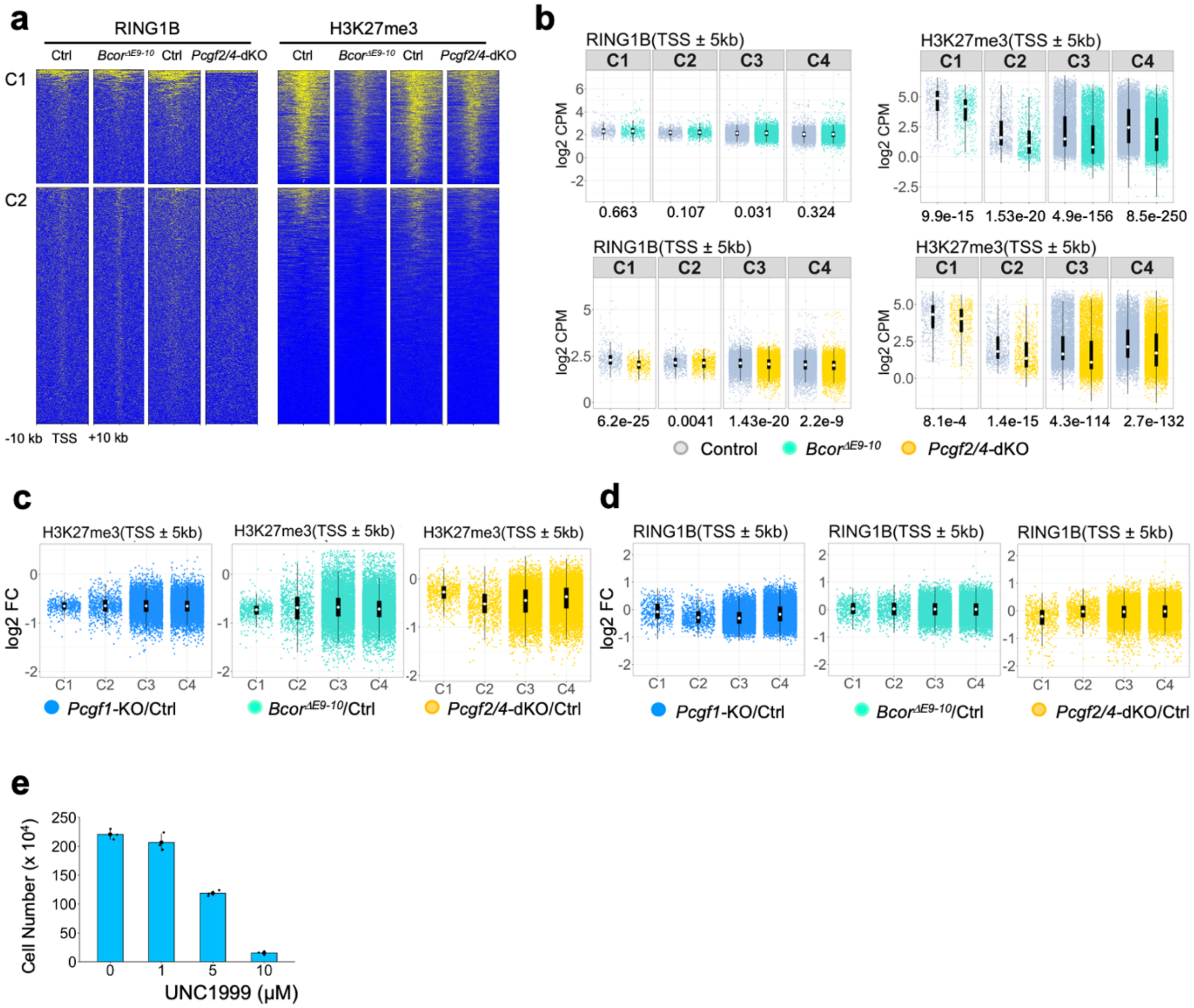

Supplementary Fig. 4 (Related to Fig. 4). ***Bcor*** $^{\Delta E9\text{-}10}$ **and *Ring1a/b*-dKO resulted in decrease of H3K27me3 enrichment while *Pcgf2*/4-dKO did not in IdHPCs.**

(a)(b)(c)(d) Contribution of PCGF1-PRC1 to stabilize H3K27me3 enrichment at promoters of C1 genes. **(a)** Heatmap views of ChIP-seq signals for RING1B and H3K27me3 across TSS (±10 kb) regions of C1 and C2 genes in control, *Bcor* $^{\Delta E9\text{-}10}$ and *Pcgf2/4*-dKO IdHPCs. Representative data of biological duplicates are shown. (**b**) Box plots for ChIP-seq signals across TSS (±5kb) for RING1B and H3K27me3 in each cluster in control, *Bcor* $^{\Delta E9\text{-}10}$ and *Pcgf2/4*-dKO IdHPCs. The numbers beneath the graph are *p*-values between the control and *Pcgf1*-KO calculated with the Wilcoxon signed-rank test. CPM: Counts Per Million. **(c)** Box plots showing the fold change of ChIP-seq signals for H3K27me3 in *Pcgf1*-KO*, Bcor* $^{\Delta E9\text{-}10}$ and *Pcgf2/4*-dKO IdHPCs. **(d)** Box plots showing the fold change of ChIP-seq signals for RING1B in *Pcgf1*-KO*, Bcor* $^{\Delta E9\text{-}10}$ and *Pcgf2/4*-dKO IdHPCs. Data in **(b), (c), and (d)** represent the means of two biologically independent analyses. The center circle indicates a median value and the boxes indicate the 25th to 75th percentile. Each dot represents individual genes. (**e**) UNC1999 dose-dependent proliferative response of IdHPCs. UNC1999 inhibited the proliferation of ERT2-Cre *Pcgf1* $^{fl/fl}$ IdHPCs in a dose-dependent manner. 5 x 10^5 cells were cultured with the indicated concentrations of UNC1999 or vehicle (DMSO) for 4 days. Data represent the mean±SD of three biologically independent experiments.

Supplementary Figure. 5

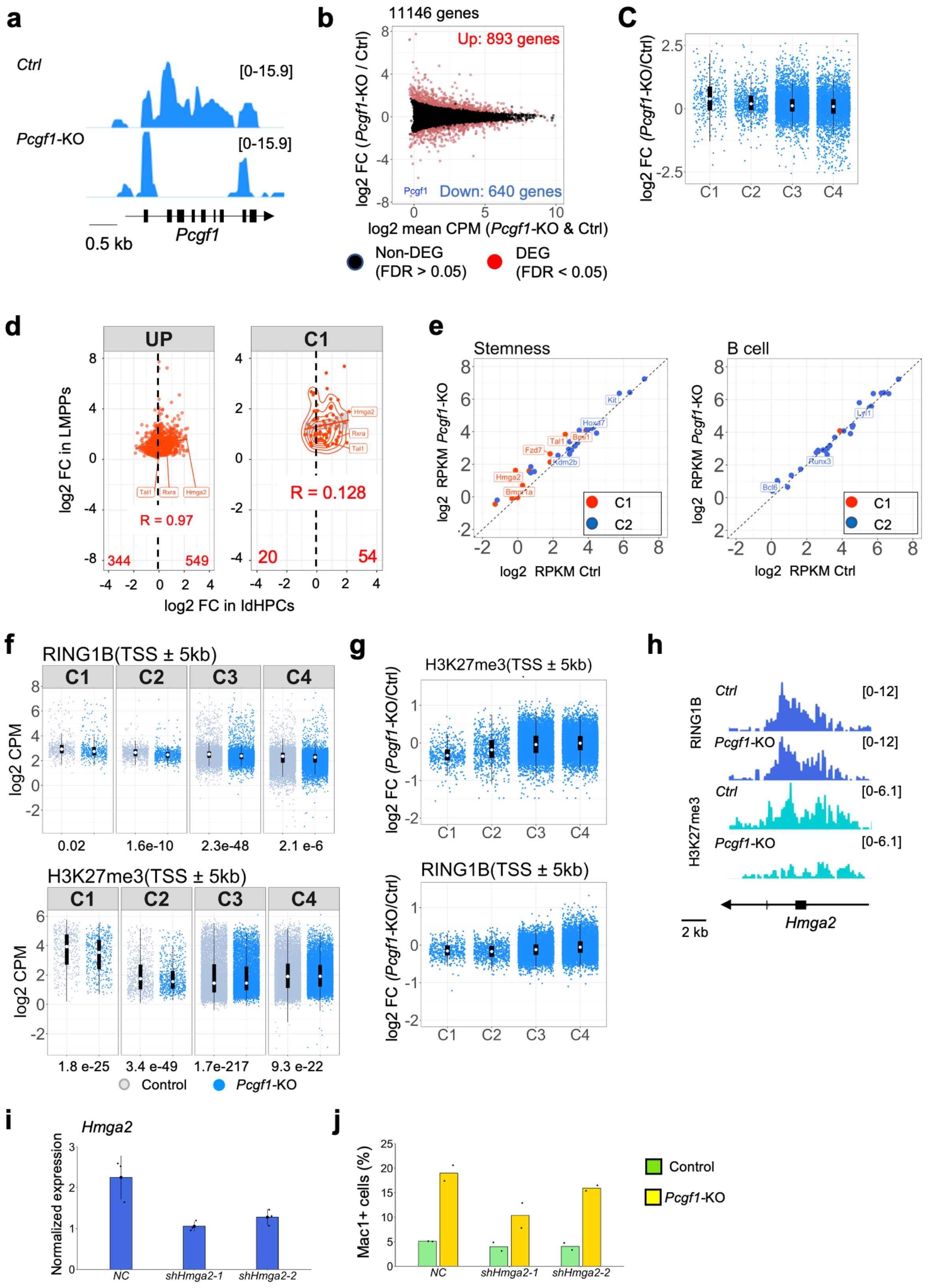

Supplementary Fig. 5 (Related to Fig. 5). PCGF1 contributes to maintaining H3K27me3 marks and repressive states in primary LMPPs, which safeguards B lineage development by silencing myeloid programs.

(**a**) Genome browser tracks showing the reduction of *Pcgf1* expression in *Pcgf1*-KO LMPPs. **(b)** An MA plot representation for DEG (red dots) between control (*Pcgf1*$^{fl/fl}$) and *Pcgf1*-KO LMPPs. Log2 fold change is plotted against the mean normalized gene expression of control and *Pcgf1*-KO LMPPs. FDR was calculated by edgeR. Low abundance genes (CPM < 1 across all samples) were filtered out. Data in the graph are the averages of biological duplicates. **(c)** Box plots showing the Log2 fold change (*Pcgf1*-KO/Control) in gene expression. The center circle indicates a median value and the boxes indicate the 25th to 75th percentile. Each dot represents individual genes. Data in graphs represent means for two biologically independent experiments. (**d**) A scatter plot showing log2 fold changes (*Pcgf1-KO*/Control) of CPM of up-regulated genes (UP) in LMPPs (Supplementary Fig. 5b) and up-regulated C1 genes (C1) in IdHPCs (X-axis) and LMPPs (Y-axis). The number in the lower side of the graphs denotes number of genes in that territory. R denotes Pearson correlation coefficient. **(e)** Gene expression profiling of "Stemness" and "B cell" related genes in control and *Pcgf1*-KO LMPPs. Data represent the average for biological duplicates. Red and blue points denote C1 and C2 genes, respectively. (**f**) Box plots showing the ChIP-seq signal across TSS (±5kb) for RING1B and H3K27me3 in each cluster in control and *Pcgf1*-KO LSK cells. **(f)** Box plots showing the fold change of ChIP-seq signals for H3K27me3 and RING1B across TSS (±5kb). Data in graphs (**f**) and (**g**) represent mean for two biologically independent experiments. The center circle indicates a median value and the boxes indicate 25th to 75th percentile. Each dot indicates individual genes. *P*-values calculated with the Wilcoxon signed rank test are indicated below each graph. H3K27me3 ChIP-seq was calibrated

by spike-in chromatin. (**h**) Screen shots for distribution of RING1B and H3K27me3 in control and *Pcgf1*-KO LSK cells around the TSS region of *Hmga2*. (**i**) Knockdown efficacy of shRNA against *Hmga2*. Relative normalized mRNA expression of *Hmga2* is shown. Data are the average of independent biological triplicates. (**j**) Bar graphs for the frequency of Mac1^{+} cells in BM cells. Bar graphs represent the mean±SD of two independent experiments.

Supplementary Figure. 6

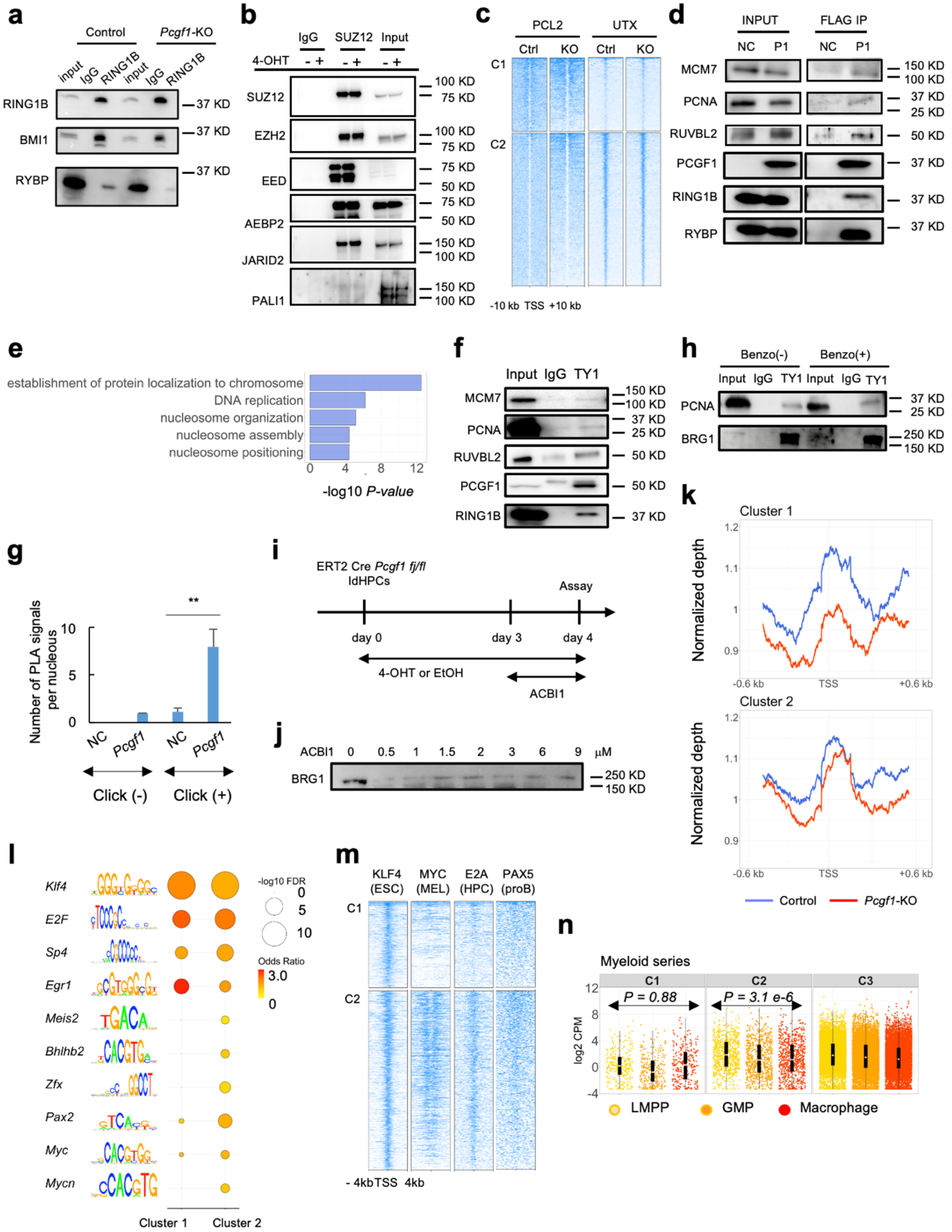

Supplementary Fig. 6 (Related to Fig. 6). PCGF1 localizes at the replication fork to affect DNA replication-linked processes.

(**a**) Immunoprecipitation by anti-RING1B and subsequent immunoblotting analysis using indicated antibodies in *Pcgf1*-KO IdHPCs. **(b)** Immunoprecipitation by anti-SUZ12 and subsequent immunoblotting analysis using antibodies for indicated antibodies in *Pcgf1*-KO IdHPCs. **(c)** Heatmap of ChIP-seq signals for PCL2 and UTX across TSS (±10 kb) regions of C1 and C2 genes in control and *Pcgf1*-KO IdHPCs. (**d**) Immunoprecipitation by FLAG-tagged PCGF1 and negative control and subsequent immunoblotting analysis using indicated antibodies in *Pcgf1*-KO IdHPCs transduced with FLAG-PCGF1 or empty vector. The data shown in the graph (**a**), (**b**), (**c**), and (**d**) are representative of two biologically independent experiments. (**e**) Selected GO terms enriched in proteins significantly associated with PCGF1 (log2 FC>0.3). (**f**) Immunoprecipitation by TY1-tagged endogenous PCGF1 and subsequent immunoblotting analysis using the indicated antibodies in *Ty1-Pcgf1* IdHPCs. The data shown in the graph are representative of biological duplicates. (**g**) Proximity ligation assay (PLA) was performed for 3x FLAG-tagged PCGF1 in EdU-labeled cells with or without the Click reaction. The bar graph indicates the quantification of the results of the PLA experiments. The data in the graph represents the mean±SD of representative of three biologically independent experiments. (**h**) Immunoprecipitation by TY1-tagged endogenous PCGF1 with or without benzonase treatment (Benzo(+) or Benzo(-), respectively) and subsequent immunoblotting analysis using indicated antibodies in *Ty1-Pcgf1* IdHPCs. The data shown in the graph are representative of biological duplicates. Benzo; benzonase. **(i)** Schematic representation of the experimental procedure. ERT2-Cre;*Pcgf1*$^{f/f}$ IdHPCs were treated with EtOH (Control) or 4-OHT (*Pcgf1*-KO) for 4 days. On day3 of the EtOH/4-OHT treatment, DMSO or 3 μM ACBl1 (MedChemExpress HY-128359) was added and incubated for 24 hours to degrade

BRG1. (j) Degradation of BRG1 by ACBI1. ERT2-Cre;*Pcgf1*$^{f/f}$ IdHPCs were treated with the indicated concentration of ACBI1 for 24 hours and immunoblotting using anti-BRG1 was performed. The data shown in the graph are representative of biological duplicates. (**k**) Meta plot showing the distribution of mean nucleosome occupancies across TSS (±0.6 kb) of Cluster 1 and 2 genes in control and *Pcgf1*-KO LSKs. The data shown in the graph are average of biological duplicates. (**l**) Results of the Motif analysis in promoters of Cluster 1 and 2 genes. (**m**) Heatmap of publicly available ChIP-seq signals for KLF4, MYC, E2A, and PAX5 across TSS (±4 kb) regions of Cluster 1 (C1) and Cluster 2 (C2) genes. (n) Box plots of gene expression in each cluster in LMPPs, GMPs, and bone marrow macrophages (GSE116177). The center white circle indicates a median value and the boxes indicate 25th to 75th percentile. Each dot represents an individual gene. The data in graphs derived from public database and are based on one biologically independent analysis. The *p*-values denote statistical significance between LMPPs and macrophages calculated with the Wilcoxon signed rank test.
