# Supplementary material for: PCGF1-PRC1 links chromatin repression with DNA replication during hematopoietic cell lineage commitment
Source: Nat Commun. 2022 Nov 28;13:7159. doi: 10.1038/s41467-022-34856-8 (PMC9705430; doi:10.1038/s41467-022-34856-8)
Supplement: Supplementary file 3 — Description of Additional Supplementary Files [file 41467_2022_34856_MOESM3_ESM.pdf]

Description of Additional Supplementary Files

Supplementary Data 1: Gene list of C1 and C2 genes.

Supplementary Data 2: Quantitative results of the PCGF1 IP-MS.

Supplementary Data 3: Quantitative results of the PCGF1 iPOND-MS.
