# Supplementary material for: PCGF1-PRC1 links chromatin repression with DNA replication during hematopoietic cell lineage commitment
Source: Nat Commun. 2022 Nov 28;13:7159. doi: 10.1038/s41467-022-34856-8 (PMC9705430; doi:10.1038/s41467-022-34856-8)
Supplement: Supplementary file 7 — Reporting Summary [file 41467_2022_34856_MOESM7_ESM.pdf]

nature research

Corresponding author(s): Haruhiko Koseki

Last updated by author(s): 2022-Oct-8

# Reporting Summary

Nature Research wishes to improve the reproducibility of the work that we publish. This form provides structure for consistency and transparency in reporting. For further information on Nature Research policies, see our Editorial Policies and the Editorial Policy Checklist.

## Statistics

For all statistical analyses, confirm that the following items are present in the figure legend, table legend, main text, or Methods section.

| n/a | Confirmed | |
|---|---|---|
| ☐ | ☒ | The exact sample size (*n*) for each experimental group/condition, given as a discrete number and unit of measurement |
| ☐ | ☒ | A statement on whether measurements were taken from distinct samples or whether the same sample was measured repeatedly |
| ☐ | ☒ | The statistical test(s) used AND whether they are one- or two-sided<br>*Only common tests should be described solely by name; describe more complex techniques in the Methods section.* |
| ☐ | ☒ | A description of all covariates tested |
| ☐ | ☒ | A description of any assumptions or corrections, such as tests of normality and adjustment for multiple comparisons |
| ☐ | ☒ | A full description of the statistical parameters including central tendency (e.g. means) or other basic estimates (e.g. regression coefficient) AND variation (e.g. standard deviation) or associated estimates of uncertainty (e.g. confidence intervals) |
| ☐ | ☒ | For null hypothesis testing, the test statistic (e.g. *F*, *t*, *r*) with confidence intervals, effect sizes, degrees of freedom and *P* value noted<br>*Give P values as exact values whenever suitable.* |
| ☒ | ☐ | For Bayesian analysis, information on the choice of priors and Markov chain Monte Carlo settings |
| ☒ | ☐ | For hierarchical and complex designs, identification of the appropriate level for tests and full reporting of outcomes |
| ☒ | ☐ | Estimates of effect sizes (e.g. Cohen's *d*, Pearson's *r*), indicating how they were calculated |

*Our web collection on statistics for biologists contains articles on many of the points above.*

## Software and code

Policy information about availability of computer code

Data collection

Flowcytometric anlysis were performed using BDFACSAria with FACSDIVA 8.0.1 (BD) software or BDFACS Calliber.
Next generation sequencing data were obtained from illumina NextSeq500. Data have been deposited and is accessible at Gene Expression Omnibus (GEO) database under accession code GSE141560.

Data analysis

FACS data were analyzed using FlowJo version 10.6.2 (BD).

Softwares and algorithms used in high throughput sequence data are follows;
HISAT2 v2.1.0
Rsubread v2.2.6
edgeR v3.30.3
cellranger v3.0.0
Seurat3.0 v4.1
Monocle v2.4.0
bowtie2 2.3.1
samtools v1.14
Picardtools v2.10.10
deeptools2 v3.3.0
pygenometracks v2.1
danpos2 v2.1.1
MACS2 v2.1.2
ChIPseeker v1.24.0

QuasR v1.28.0
clusterprofiler v 3.16.1
BSgenome v1.38.0
Biostrings v2.38.4
Ststistical analysis and visualization were conducted using R version 3.2.2 except for single cell RNA-seq analysis by Seurat and Monocle which required newer version of R and performed by R version 4.0.0. Custom codes to conduct data analysis in this paper were deposited to Zenode repository under the DOI code 10.5281/zenodo.7114888.

For manuscripts utilizing custom algorithms or software that are central to the research but not yet described in published literature, software must be made available to editors and reviewers. We strongly encourage code deposition in a community repository (e.g. GitHub). See the Nature Research guidelines for submitting code & software for further information.

## Data

Policy information about availability of data

All manuscripts must include a data availability statement. This statement should provide the following information, where applicable:

- Accession codes, unique identifiers, or web links for publicly available datasets
- A list of figures that have associated raw data
- A description of any restrictions on data availability

The high-throughput sequencing data generated in this study have been deposited in the GEO database under accession code GSE141560(https://www.ncbi.nlm.nih.gov/geo/query/acc.cgi?acc=GSE141560). The proteomic data used in this study are available in the Pride database under accession code PXD036330. Other data generated in this study are provided in the Supplementary Information/Source Data file.
Publicly available data utilized in this study are;
RNA-seq for hematopoietic progenitor cells: GSE116177 https://www.ncbi.nlm.nih.gov/geo/query/acc.cgi?acc=GSE116177
Single cell RNA-seq profiling of HPSC: GSE81682 https://www.ncbi.nlm.nih.gov/geo/query/acc.cgi?acc=GSE81682
ChIP-seq for KLF4 in ESCs: GSM1324615 https://www.ncbi.nlm.nih.gov/geo/query/acc.cgi?acc=GSM1324615
ChIP-seq for MYC in MEL cells: GSM912934 https://www.ncbi.nlm.nih.gov/geo/query/acc.cgi?acc=GSM912934
ChIP-seq for E2A in HPCs: GSM546535 https://www.ncbi.nlm.nih.gov/geo/query/acc.cgi?acc=GSM546535
ChIP-seq for PAX5 in HPCs: GSM2863171 https://www.ncbi.nlm.nih.gov/geo/query/acc.cgi?acc=GSM2863171
Mouse reference genome mm9: https://genome.ucsc.edu/cgi-bin/hgGateway?db=mm9

# Field-specific reporting

Please select the one below that is the best fit for your research. If you are not sure, read the appropriate sections before making your selection.

[x] Life sciences   [ ] Behavioural & social sciences   [ ] Ecological, evolutionary & environmental sciences

For a reference copy of the document with all sections, see nature.com/documents/nr-reporting-summary-flat.pdf

# Life sciences study design

All studies must disclose on these points even when the disclosure is negative.

| | |
|---|---|
| Sample size | Sample size was chosen to be large enough for each condition and biological replicates according to standards of the field. In case of ChIP-seq, measurement performed on two independent biological replicates are set to be standard by ENCODE Consortium (Landt, Stephen G et al. Genome research vol. 22,9 (2012): 1813-31) and we've followed this guideline. Similarly, we've also conducted RNA-seq and MNase-seq on two independent biological replicates and reproducibility of these sequencing data was confirmed by analysis including PCA. In other experiments we've conducted basically three (at least two) independent biological replicates. Sample-size was determined to be adequate based on the consistency of the results. |
| Data exclusions | No data were excluded from the analysis. |
| Replication | Recorded results were consistently replicated. ChIP-seq were performed 2 independent times according to the ENCODE guideline and other experiments were performed more than 2 to 3 times as noted in figures. |
| Randomization | Mice used in experiments were matched in age and sex. |
| Blinding | Blinding were not performed because investigators needed to know the treatment group. As our results is based on objective measurements but not on human observation, we've avoided the bias. |

# Reporting for specific materials, systems and methods

We require information from authors about some types of materials, experimental systems and methods used in many studies. Here, indicate whether each material, system or method listed is relevant to your study. If you are not sure if a list item applies to your research, read the appropriate section before selecting a response.

## Materials & experimental systems

| n/a | Involved in the study | |
|---|---|---|
| | x | Antibodies |
| x | | Eukaryotic cell lines |
| x | | Palaeontology and archaeology |
| | x | Animals and other organisms |
| x | | Human research participants |
| x | | Clinical data |
| x | | Dual use research of concern |

## Methods

| n/a | Involved in the study | |
|---|---|---|
| | x | ChIP-seq |
| | x | Flow cytometry |
| x | | MRI-based neuroimaging |

# Antibodies

Antibodies used

anti m2_FLAG(Sigma-Aldrich, F3165) https://www.labome.com/product/Sigma-Aldrich/F3165.html
anti Ubiquityl-Histone H2A (Lys119)(D27C4) (Cell Signaling, #8240) https://www.cellsignal.jp/products/primary-antibodies/ubiquityl-histone-h2a-lys119-d27c4-xp-rabbit-mab/8240
anti SUZ12(D39F6) (Cell Signaling #3737) https://www.cellsignal.jp/products/primary-antibodies/suz12-d39f6-xp-rabbit-mab/3737
anti H3K27me3 (Merck Millipore, 07-449) https://www.merckmillipore.com/JP/ja/product/Anti-trimethyl-Histone-H3-Lys27-Antibody,MM_NF-07-449
anti-acetyl-Histone H3(Lys27) (Merk Millipore, 07-360) https://www.merckmillipore.com/JP/ja/product/Anti-acetyl-Histone-H3-Lys27-Antibody,MM_NF-07-360
anti-RNA polymerase II CTD repeat YSPTSPS [8WG16] - ChIP Grade (abcam, ab817) https://www.citeab.com/antibodies/752154-ab817-anti-rna-polymerase-ii-ctd-repeat-ysptsps-antib
anti Bmi1 (D20B7) XP Rabitt mAb (Cell Signaling, #6964) https://www.cellsignal.jp/products/primary-antibodies/bmi1-d20b7-xp-rabbit-mab/6964
anti PCGF1(E-8)(Santa Cruz Biotechnology , sc-515371) https://www.scbt.com/p/pcgf1-antibody-e-8?productCanUrl=pcgf1-antibody-e-8&_requestid=2190661
anti EZH2(D2C9) (Cell Signaling, #5246) https://www.cellsignal.jp/products/primary-antibodies/ezh2-d2c9-xp-rabbit-mab/5246
anti AEBP2(D7C6x) (Cell Signaling, #14129) https://en.cellsignal.jp/products/primary-antibodies/aebp2-d7c6x-rabbit-mab/14129
anti JARID2 (D6M9X)(Cell Signaling, #13594) https://en.cellsignal.jp/products/primary-antibodies/jarid2-d6m9x-rabbit-mab/13594
anti DEDAF Merck Millipore, AB3637) https://www.merckmillipore.com/JP/ja/product/Anti-DEDAF-Antibody,MM_NF-AB3637
anti SKP1(D3J4N) (Cell Signaling, #12248) https://en.cellsignal.jp/products/primary-antibodies/skp1-d3j4n-rabbit-mab/12248
anti MCM7(141.2)(Santa Cruz Biotechnology, sc-9966) https://www.scbt.com/p/mcm7-antibody-141-2?productCanUrl=mcm7-antibody-141-2&_requestid=1746485
anti PCNA(PC10) (Santa Cruz Biotechnology, sc-56) https://www.scbt.com/p/pcna-antibody-pc10?productCanUrl=pcna-antibody-pc10&_requestid=1748097
anti RUVBL2 （Bethyl Laboratories, A302-536A） https://www.fortislife.com/products/primary-antibodies/rabbit-anti-ruvbl2-antibody/BETHYL-A302-536
anti BCOR (Proteintech, 12107-1-AP) https://www.ptglab.co.jp/products/BCOR-Antibody-12107-1-AP.htm
anti TY1 (Rockland Immunochemicals Inc. 200-301-W45) https://www.rockland.com/categories/primary-antibodies/ty1-antibody-200-301-W45/
anti SMARCA4/BRG1 (Proteintech, 21634-1-AP) https://www.ptglab.com/products/SMARCA4-Antibody-21634-1-AP.htm
Anti UTX(Merck Millipore, ABE1865) https://www.merckmillipore.com/JP/ja/product/Anti-UTX-KDM6A,MM_NF-ABE1865
anti RING1B (in house)
anti PHC2 (in house)
anti EED (in house)
anti KDM2B (in house)
Anti-Mouse IgG, HRP-Linked Whole Ab Sheep(Cytiva, NA931) https://www.cytivalifesciences.co.jp/catalog/0428.html
Anti-Rabbit IgG, HRP-Linked Whole Ab Donkey(Cytiva, NA934) https://www.cytivalifesciences.co.jp/catalog/0428.html
APC Rat anti Mouse CD45.1 (30-F11) (BD 559864)
PE-Cy7 Rat anti Mouse CD117 (2B8) (BD 558163)
APC Rat anti Mouse CD117 (2B8) (BD BD561074)
PE Rat anti Mouse Ly6A/E (D7)(BD BD562059)
APC-Cy7 Rat anti Mouse Ly6A/E (D7)(BD BD560654)
PE Rat anti Mouse CD135 (A2F10.1)(BD 553842)
CD34 Monoclonal Antibody (RAM34), FITC, eBioscience™ (Invitrogen 11-0341-81)
APC Rat anti Mouse CD127 (SB/199) (BD 564175)
APC anti-mouse CD93 (AA4.1, early B lineage) Antibody (C1qRp) (Biolegend 136509)
PE Rat anti Mouse CD19 (1D3) (BD 553786)
APC/Cy7 Rat anti Mouse B220 (RA3-6B2) (BD 552772)
PE/Cy7 Rat anti Mouse IgM (R6-60.2) (BD 552867)
BV421 Rat anti Mouse CD43 (S7) (BD 752957)
PE Rat anti Mouse IgD (11-26C) (BD 558597)
APC/Cy7 Rat anti Mouse CD8 (53-6.7) (BD 561967)
BV421 Rat anti Mouse CD4 (H129.19) (BD 740024)
BV510 Hamster anti Mouse CD3ε (145-2C11) (BD 563024)

FITC Rat anti Mouse CD25 (7D4) (BD 553071)
PE Rat anti Mouse CD44 (IM7) (BD 553134)
PerCP-Cy5.5 Mouse anti Mouse NK1.1 (PK136) (BD 561111)
PerCP-Cy5.5 Mouse Lineage Antibody Cocktail, with Isotype Control (BD 561317)
PE-Cy7 Rat Anti-Mouse TER-119/Erythroid Cells TER-119 (BD 557853)
FITC Rat anti Mouse Mac-1 (M1/70) (BD 557396)
V450 Rat anti Mouse Mac-1 (M1/70) (BD 560456)
PE Mouse anti Human CD25 (M-A251) (BD 55432)

Validation

Validation of species and applications of all commercially available antibodies are shown by manufactures in the above listed websites. Antibody for RING1B, PHC2 and KDM2B are laboratory made. The quality of these antibodies were validated in Blackledge, N. P. et al. Cell 157, 1445–1459 (2014), Endoh, M. et al. PLoS Genet. 8, (2012), and Sugishita, H. et al. Nat. Commun. 12, 5341 (2021)..

# Animals and other organisms

Policy information about studies involving animals; ARRIVE guidelines recommended for reporting animal research

Laboratory animals

C57BL/6 (B6) mice were purchased from CLEA Japan Inc. C57BL/6-Ly5.1 mice were purchased from Charles River Japan. ERT2-Cre Pcgf1 fl/fl, ERT2-Cre Pcgf2/4 fl/flTy1-Pcgf1, ERT2-Cre Ring1a -/-Ring1b fl/fl, and ERT2-Cre mice were generated and maintained in our animal facility. Bcor mutant mice was generated and provided by Vivian J. Bardwell. Female mIce of 6-8 weeks of age were used. For the analysis of hematopoietic cells derived from fetal livers, fetuses at 14 days post-coitum (dpc) were obtained by timed mating. The day that a plug was observed was referred to as 0 dpc. The housing conditions are as follows. Light cycle: A 14-hour light/10-hour cycle. Temperature and humidity: 18-23C with 40-60% humidity.

Wild animals

The study did not involve wild animals.

Field-collected samples

The study did not involve field-collected samples.

Ethics oversight

All experiments were conducted according to guidelines approved by the Institutional Animal Care and Use Committee of RIKEN's Yokohama office.

Note that full information on the approval of the study protocol must also be provided in the manuscript.

# ChIP-seq

## Data deposition

☒ Confirm that both raw and final processed data have been deposited in a public database such as GEO.

☒ Confirm that you have deposited or provided access to graph files (e.g. BED files) for the called peaks.

Data access links
*May remain private before publication.*

All ChIP-seq data (rawdata and bigwig files) have been deposited in the GEO database (The accession number is GSE141560).

Files in database submission

GSM4206934 Pcgf1_f/f_input_1
GSM4206935 Pcgf1_f/f_input_2
GSM4206936 Pcgf1_-/-_input 1
GSM4206937 Pcgf1_-/-_input 2
GSM4206938 NC_for_Pcgf1
GSM4206939 FLAG_PCGF1_1
GSM4206940 FLAG_PCGF1_2
GSM4206941 Pcgf1_f/f_RING1B_1
GSM4206942 Pcgf1_f/f_RING1B_2
GSM4206943 Pcgf1_-/-_RING1B_1
GSM4206944 Pcgf1_-/-_RING1B_2
GSM4206945 Ring1A/B_-/-_RING1B
GSM4206952 Pcgf1_f/f_SUZ12_1
GSM4206953 Pcgf1_f/f_SUZ12_2
GSM4206954 Pcgf1_-/-_SUZ12_1
GSM4206955 Pcgf1_-/-_SUZ12_2
GSM4206956 Pcgf1_f/f_H3K27me3_spike_in_1
GSM4206957 Pcgf1_f/f_H3K27me3_spike_in_2
GSM4206958 Pcgf1_-/-_H3K27me3_spike_in_1
GSM4206959 Pcgf1_-/-_H3K27me3_spike_in_2
GSM4206960 Pcgf1_f/f_H3K27ac_1
GSM4206961 Pcgf1_f/f_H3K27ac_2
GSM4206962 Pcgf1_f/f_H3K27me3_1
GSM4206963 Pcgf1_f/f_H3K27me3_2
GSM4206964 Pcgf1_-/-_H3K27me3_1
GSM4206965 Pcgf1_-/-_H3K27me3_2

GSM4206966 Pcgf1_f/f_Pol2_1
GSM4206967 Pcgf1_f/f_Pol2_2
GSM4206968 Pcgf1_f/f_PHC2_1
GSM4206969 Pcgf1_f/f_PHC2_2
GSM4206970 Pcgf1_-/-_PHC2_1
GSM4206971 Pcgf1_-/-_PHC2_2
GSM4206972 Pcgf1_f/f_H2A.Z_1
GSM4206973 Pcgf1_f/f_H2A.Z_2
GSM4206974 Pcgf1_-/-_H2A.Z_1
GSM4206975 Pcgf1_-/-_H2A.Z_2
GSM4206980 LSK_Pcgf1_f/f_H3K27me3_spike_in_1
GSM4206981 LSK_Pcgf1_f/f_H3K27me3_spike_in_2
GSM4206982 LSK_Pcgf1_-/-_H3K27me3_spike_in_1
GSM4206983 LSK_Pcgf1_-/-_H3K27me3_spike_in_2
GSM4206984 LSK_Pcgf1_f/f_RING1B_1
GSM4206985 LSK_Pcgf1_f/f_RING1B_2
GSM4206986 LSK_Pcgf1_-/-_RING1B_1
GSM4206987 LSK_Pcgf1_-/-_RING1B_2
GSM6310957 Pcgf1_f/f_H3K27me3_1, chIP
GSM6310958 Pcgf1_f/f_H3K27me3_2, chIP
GSM6310959 Pcgf1_-/-_H3K27me3 1
GSM6310960 Pcgf1_-/-_H3K27me3 2
GSM6310961 Pcgf1_f/f_BCOR_1
GSM6310962 Pcgf1_f/f_BCOR_2
GSM6310963 Pcgf1_-/-_BCOR 1
GSM6310964 Pcgf1_-/-_BCOR 2
GSM6310965 Pcgf1_f/f_UTX_1
GSM6310966 Pcgf1_f/f_UTX_2
GSM6310967 Pcgf1_-/-_UTX_1
GSM6310968 Pcgf1_-/-_UTX_2
GSM6310969 Pcgf1_f/f_PCL2_1
GSM6310970 Pcgf1_f/f_PCL2_2
GSM6310971 Pcgf1_-/-_PCL2_1
GSM6310972 Pcgf1_-/-_PCL2_2
GSM6310973 Pcgf1_f/f_H2AK119ub_1
GSM6310974 Pcgf1_f/f_H2AK119ub_2
GSM6310975 Pcgf1_-/-_H2AK119ub_1
GSM6310976 Pcgf1_-/-_H2AK119ub_2
GSM6310977 Negative control for Ty1-Pcgf1_1
GSM6310978 Negative control for Ty1-Pcgf1_2
GSM6310979 Ty1 Pcgf1 (Bulk)_1
GSM6310980 Ty1 Pcgf1 (Bulk)_2
GSM6310981 Ty1 Pcgf1 (Nascent DNA)_1
GSM6310982 Ty1 Pcgf1 (Nascent DNA)_2
GSM6310983 BCOR delta E9-10 H3K27me3 1
GSM6310984 BCOR delta E9-10 H3K27me3 2
GSM6310985 BCOR WT H3K27me3 1
GSM6310986 BCOR WT H3K27me3 2
GSM6310987 Pcgf2/4 f/f H3K27me3 1
GSM6310988 Pcgf2/4 f/f H3K27me3 2
GSM6310989 Pcgf2/4 -/- H3K27me3 1
GSM6310990 Pcgf2/4 -/- H3K27me3 2
GSM6310991 BCOR WT RING1B 1
GSM6310992 BCOR WT RING1B 2
GSM6310993 BCOR delta E9-10 RING1B 1
GSM6310994 BCOR delta E9-10 RING1B 2
GSM6310995 Pcgf2/4 f/f RING1B 1
GSM6310996 Pcgf2/4 f/f RING1B 2
GSM6310997 Pcgf2/4 -/- RING1B 1
GSM6310998 Pcgf2/4 -/- RING1B 2
GSM6311015 Pcgf2/4 f/f PHC2 1
GSM6311016 Pcgf2/4 f/f PHC2 2
GSM6311017 Pcgf2/4 -/- PHC2 1
GSM6311018 Pcgf2/4 -/- PHC2 2
GSM6311019 Pcgf1_f/f_KDM2B_1
GSM6311020 Pcgf1_f/f_KDM2B_2
GSM6311021 Pcgf1_-/-_KDM2B_1
GSM6311022 Pcgf1_-/-_KDM2B_2

GSM6311023 Pcgf1_f/f_H2AK119ub_1 [FC00472]
GSM6311024 Pcgf1_f/f_H2AK119ub_2 [FC00472]
GSM6311025 Pcgf1_-/-_H2AK119ub_1 [FC00472]
GSM6311026 Pcgf1_-/-_H2AK119ub_2 [FC00472]
GSM6311027 Ring1a -/- 1b -/- H2AK119ub 1
GSM6311028 Ring1a -/- 1b -/- H2AK119ub 2
GSM6311029 Pcgf1_f/f_JARID2_1
GSM6311030 Pcgf1_f/f_JARID2_2
GSM6311031 Pcgf1_-/-_JARID2_1
GSM6311032 Pcgf1_-/-_JARID2_2
GSM6311033 ESC RING1B
GSM6311034 ESCSUZ12

Genome browser session (e.g. UCSC): IGV

## Methodology

Replicates: Two replicates for all ChIP-seq data excluding S09955.fastq.gz. Details are described in GEO meta data sheet.

Sequencing depth: Between 10 to 50 million reads per samples.

Antibodies:
anti m2_FLAG(Sigma-Aldrich, F3165) https://www.labome.com/product/Sigma-Aldrich/F3165.html
anti Ubiquityl-Histone H2A (Lys119)(D27C4) (Cell Signaling, #8240) https://www.cellsignal.jp/products/primary-antibodies/ubiquityl-histone-h2a-lys119-d27c4-xp-rabbit-mab/8240
anti SUZ12(D39F6) (Cell Signaling #3737) https://www.cellsignal.jp/products/primary-antibodies/suz12-d39f6-xp-rabbit-mab/3737
anti H3K27me3 (Merck Millipore, 07-449) https://www.merckmillipore.com/JP/ja/product/Anti-trimethyl-Histone-H3-Lys27-Antibody,MM_NF-07-449
anti-acetyl-Histone H3(Lys27) (Merk Millipore, 07-360) https://www.merckmillipore.com/JP/ja/product/Anti-acetyl-Histone-H3-Lys27-Antibody,MM_NF-07-360
anti-RNA polymerase II CTD repeat YSPTSPS [8WG16] - ChIP Grade (abcam, ab817) https://www.citeab.com/antibodies/752154-ab817-anti-rna-polymerase-ii-ctd-repeat-ysptsps-antib
anti H2A.Z- ChIP Grade (abcam, ab4174) https://www.citeab.com/antibodies/764060-ab4174-anti-histone-h2a-z-antibody-chip-grade
anti EZH2(D2C9) (Cell Signaling, #5246) https://www.cellsignal.jp/products/primary-antibodies/ezh2-d2c9-xp-rabbit-mab/5246
anti BCOR (Proteintech, 12107-1-AP) https://www.ptglab.co.jp/products/BCOR-Antibody-12107-1-AP.htm
anti TY1 (Rockland Immunochemicals Inc. 200-301-W45) https://www.rockland.com/categories/primary-antibodies/ty1-antibody-200-301-W45/
anti SMARCA4/BRG1 (Proteintech, 21634-1-AP) https://www.ptglab.com/products/SMARCA4-Antibody-21634-1-AP.htm
Anti UTX(Merck Millipore, ABE1865) https://www.merckmillipore.com/JP/ja/product/Anti-UTX-KDM6A,MM_NF-ABE1865
anti RING1B (in house)
anti PHC2 (in house)
anti KDM2B (in house)

Peak calling parameters: Mapping was carried out by bowtie2 with default setting. De-duplication was performed using Picardtools (http://broadinstitute.Github.io/picard). Peak calling were performed by MACS2 or DANPOS2 . Regions of H3K27me3 enrichment were identified using the dpeak function of DANPOS2(-q 40 -kw 750 -kd 1500, height_logP > 120) . PCGF1 and RING1B peaks were generated using MACS2 broad mode

Data quality: We've visually confirmed the quality of ChIP-seq by comparing the distribution of ChIP fragments around TSS against negative control in the form of Heatmap generated by deeptools2.

Software:
bowtie2 2.3.1
samtools v1.14
Picardtools v2.10.10
deeptools2 v3.3.0
pygenometracks v2.1
danpos2 v2.1.1
MACS2 v2.1.2
ChIPseeker v1.24.0
QuasR v1.28.0
clusterprofiler v 3.16.1

# Flow Cytometry

## Plots

Confirm that:

- [x] The axis labels state the marker and fluorochrome used (e.g. CD4-FITC).
- [x] The axis scales are clearly visible. Include numbers along axes only for bottom left plot of group (a 'group' is an analysis of identical markers).
- [x] All plots are contour plots with outliers or pseudocolor plots.
- [x] A numerical value for number of cells or percentage (with statistics) is provided.

## Methodology

| | |
|---|---|
| Sample preparation | After the harvest of the cells, the cell concentration was adjusted to under 1 x 107 cells per 100 ml with Minimum Essential Media (MEM) (ThermoFisher 11095080) supplemented with 1% Fetal Bovine Serum (FBS) and NaHCO3. Then, antibodies were added and incubated on ice in the dark for 30 min. Cells were washed with 1 ml MEM supplemented with 1% FBS and NaHCO3. Precipitated cells were suspended in 1 ml ice-cold MEM supplemented with 1% FBS and NaHCO3 and filtered through a 37 mm nylon mesh and then run FACS (BDFACSAria with FACSDIVA 8.0.1 (BD) software were used when cells derived from BMT experiments were analyzed and when cells derived from in vitro culture were analyzed BDFACS Calliber was used.). |
| Instrument | The data was collected on a BD Aria or Calliber flow cytometer. |
| Software | All flow data was collected using FACSDIVA 8.0.1 (BD) and analyzed using FlowJo version 10.6.2 (BD). |
| Cell population abundance | Ten-thousands cells (LSK cells) and 2000 cells (LMPPs) were acquired from one mouse. The percentage of each population are indicated in the figures. Purity of the populations were validated by a post sort analysis by FACS. |
| Gating strategy | Total cell population was first plotted for SSC-A and FSC-A to gate cells from debris. Next, FSC-H/FSC-W and SSC-H/SSC-W were used to remove doublet cells. Regarding the boundary of the positive and negative staining cell population, we usually regarded 103 (Aria) and 101 (Calliber) as a boundary, however unlabeled controls were also used to determine the boundaries between positive and negative staining cell population. Gating strategies are provided in Extended Data Fig. 1 and Fig. 4. |

- [x] Tick this box to confirm that a figure exemplifying the gating strategy is provided in the Supplementary Information.
