# Supplementary material for: PCGF1-PRC1 links chromatin repression with DNA replication during hematopoietic cell lineage commitment
Source: Nat Commun. 2022 Nov 28;13:7159. doi: 10.1038/s41467-022-34856-8 (PMC9705430; doi:10.1038/s41467-022-34856-8)
Supplement: Supplementary file 8 — Source Data [file 41467_2022_34856_MOESM8_ESM.zip › SorurceData/uncropped images of gels and blots..pdf]

Figure 1. b

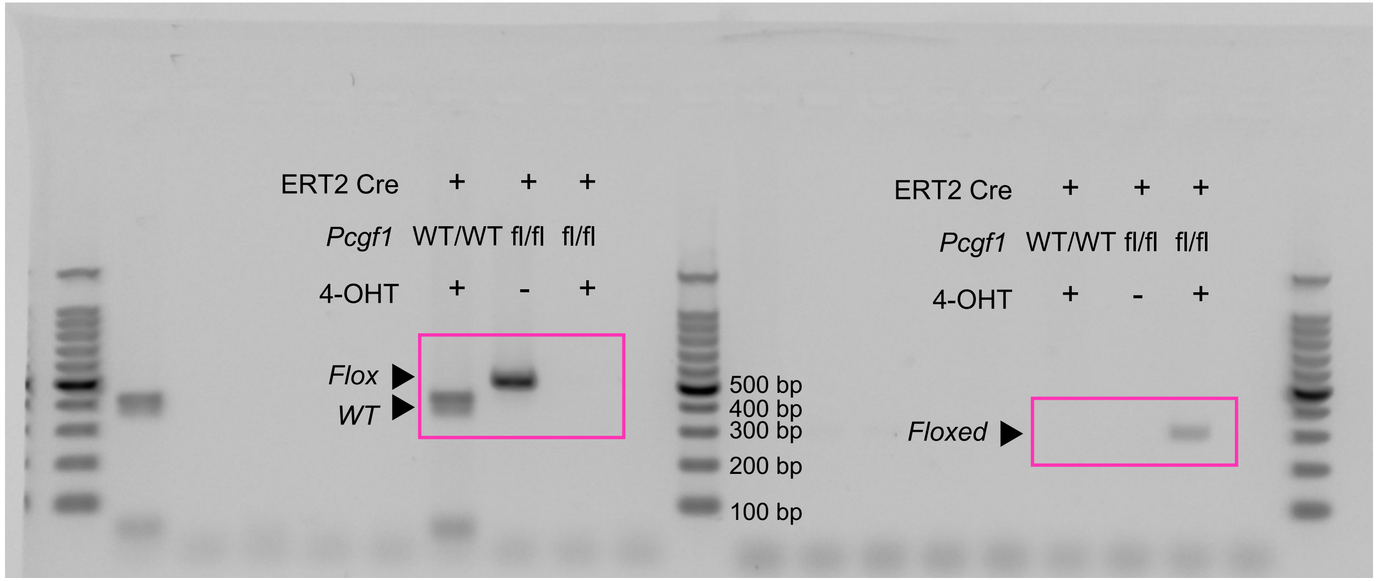

Supplementary Fig 2. i

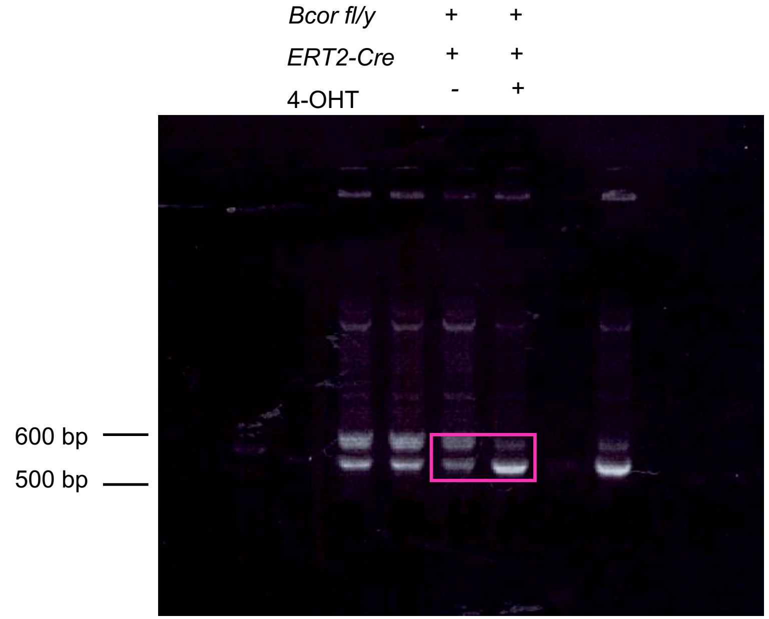

Figure 2. h

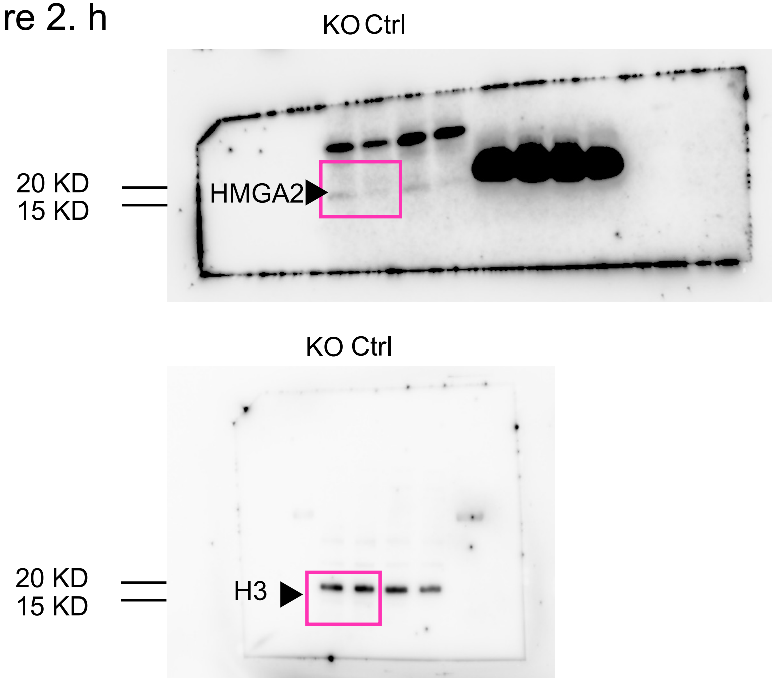

Supplementary Fig 2. b

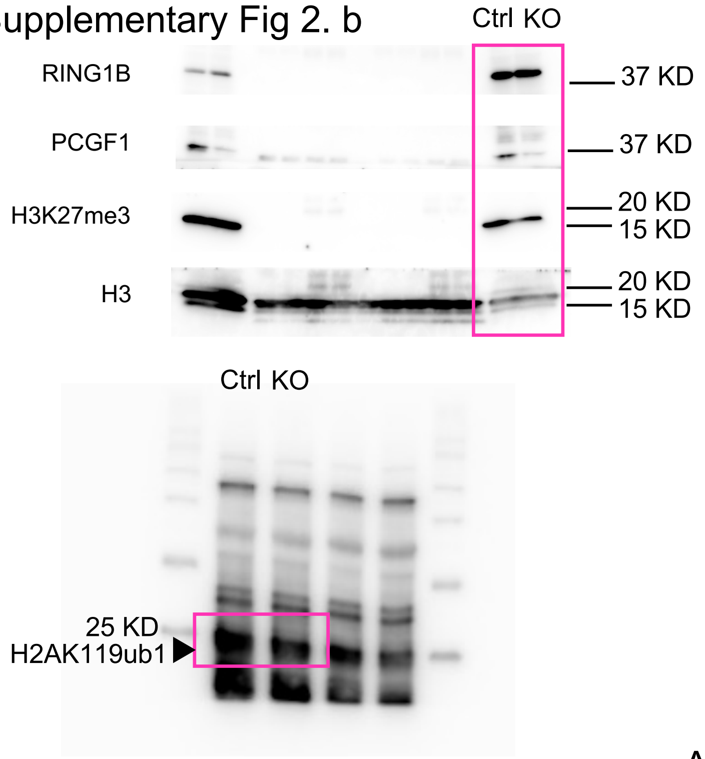

Cttl : Control

KO : *Pcgf1*-KO

Supplementary Fig 2. j

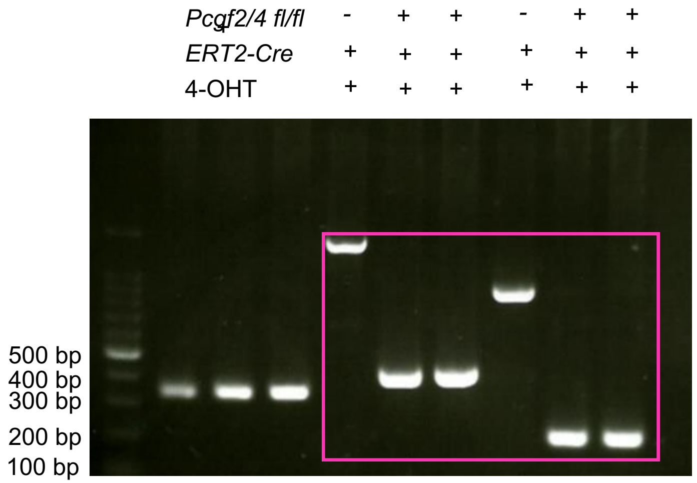

Areas shown in Figures are highlighted by pink squares.

Supplementary Fig 6. a

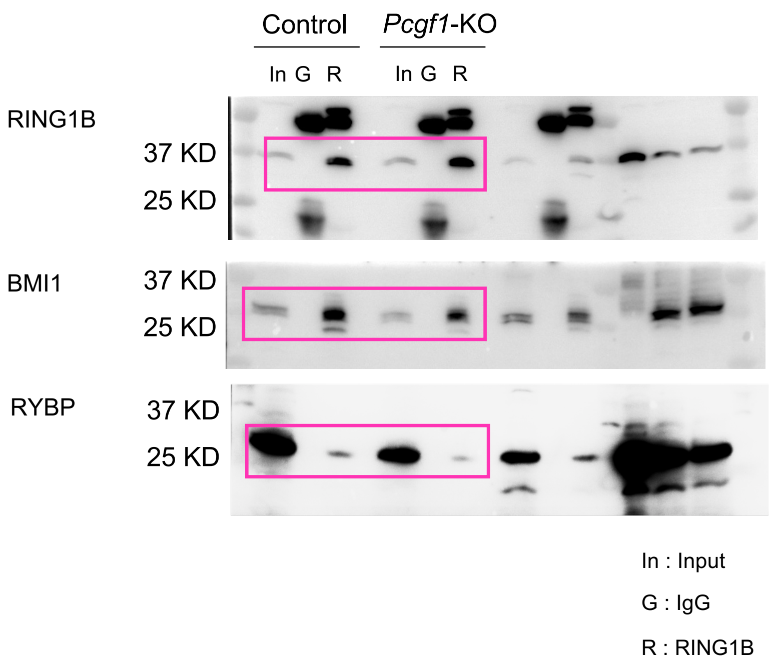

Supplementary Fig 6. b

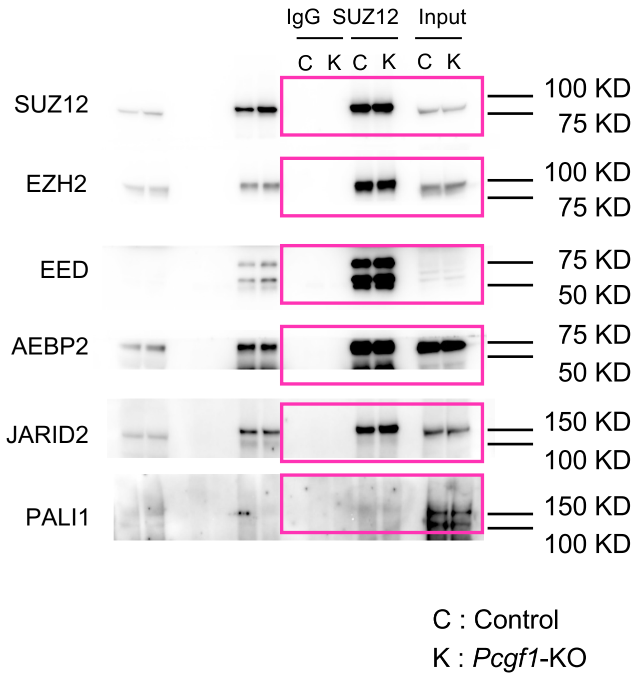

Supplementary Fig 6. d

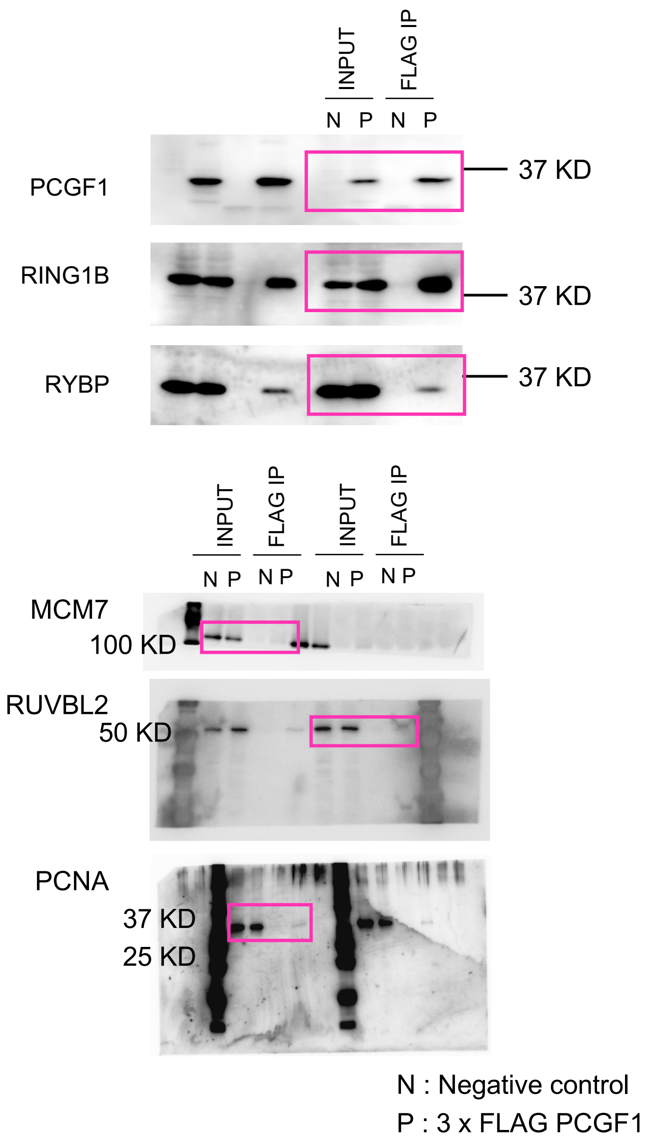

Areas shown in Figures are highlighted by pink squares.

Figure 6. e

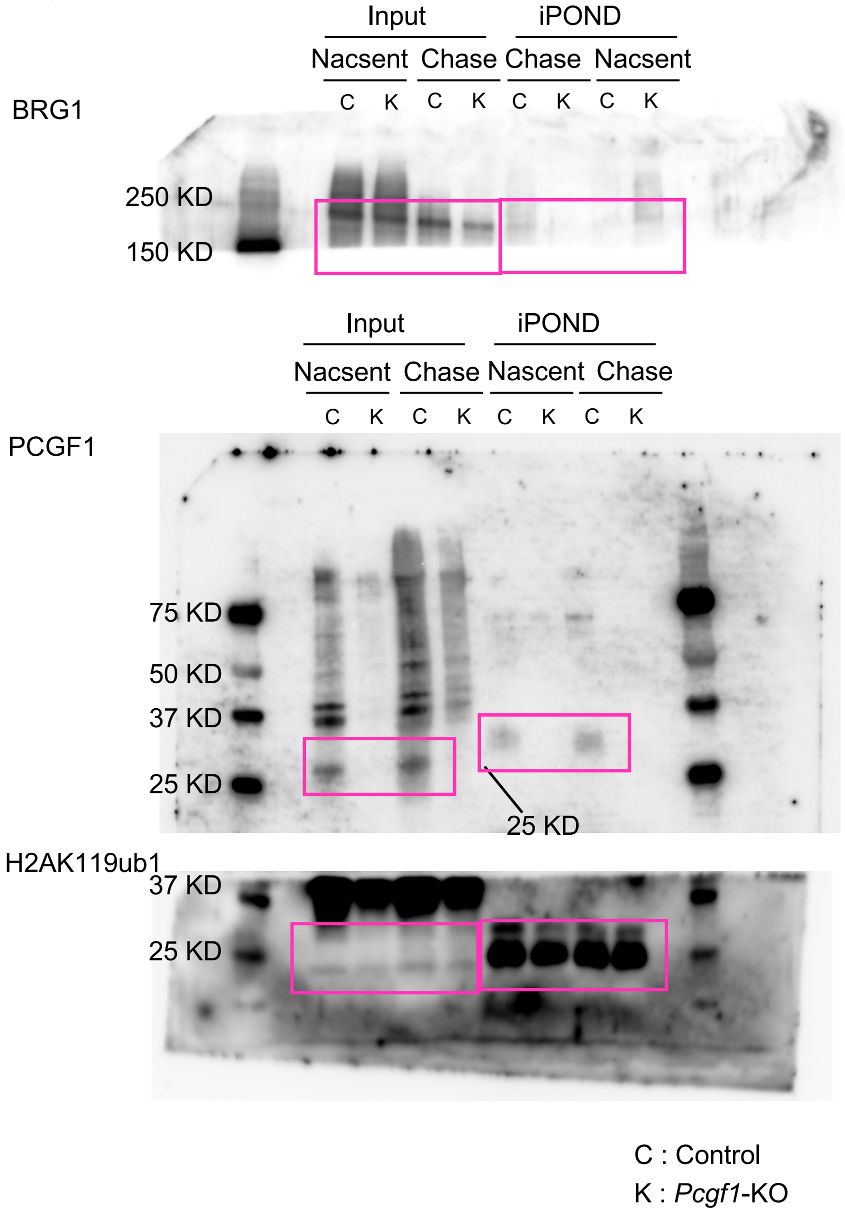

Supplementary Fig 6. f

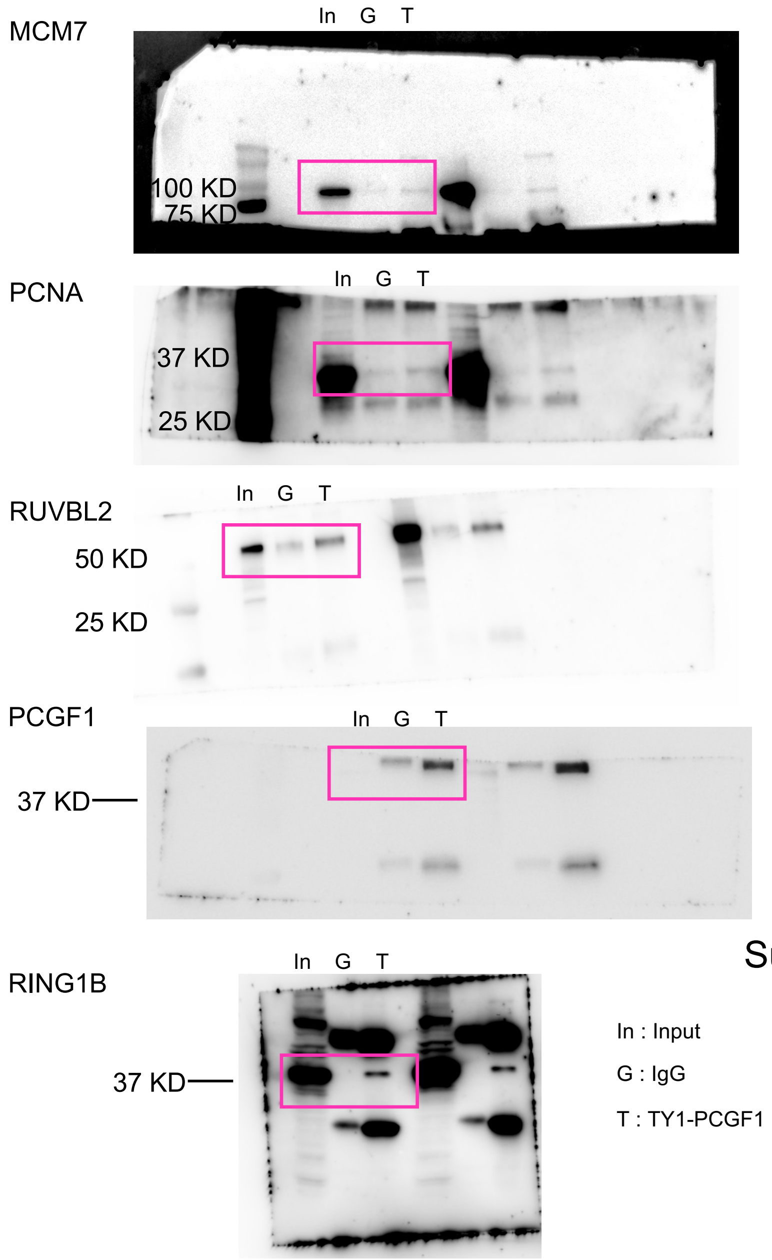

Supplementary Fig 6. h

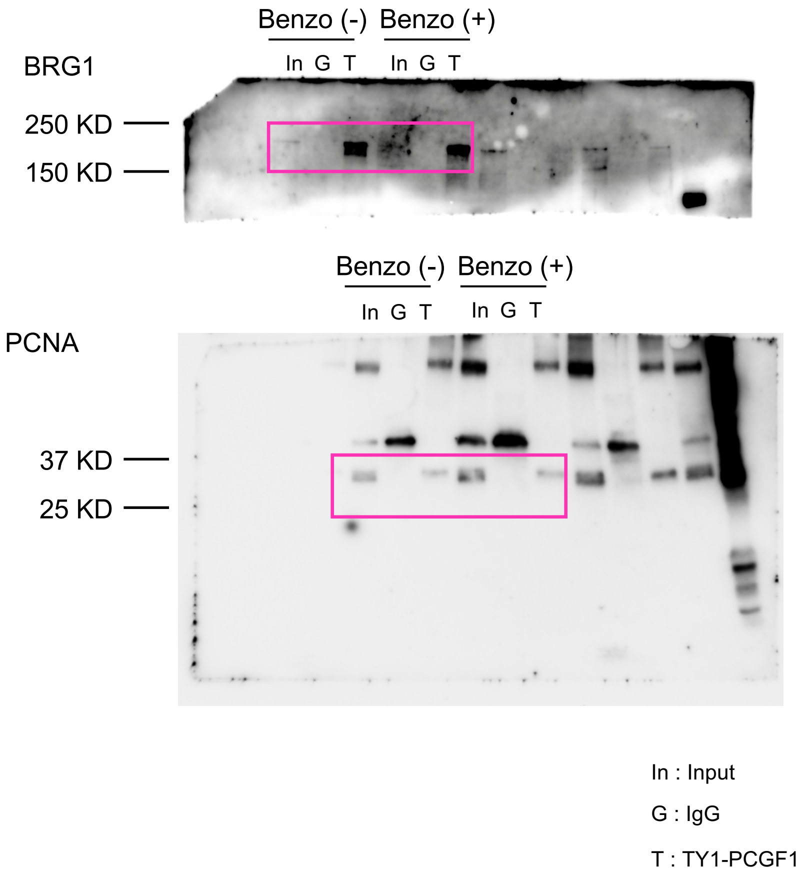

Supplementary Fig 6. j

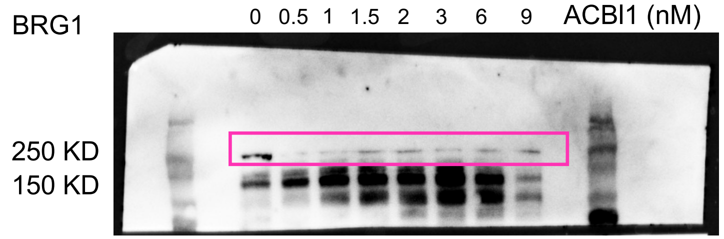

Areas shown in Figures are highlighted by pink squares.
